# Successful treatment of a gravida with acute type A aortic dissection in the third trimester: A case report

**DOI:** 10.1097/MD.0000000000033423

**Published:** 2023-03-31

**Authors:** Yue Wang, Jianmao Hong, Bijun Xu, Shiqiang Wang, Fan He

**Affiliations:** a Department of Cardiovascular Surgery, Sir Run Run Shaw Hospital, Zhejiang University, Zhejiang, China.

**Keywords:** multidisciplinary consultation, pregnant, type A aortic dissection

## Abstract

Acute type A aortic dissection is a rare and catastrophic complication of pregnancy with a very high mortality rate for both the mother and the fetus.

**Patient concerns:**

A 40-year-old female who was 31 weeks pregnant was transferred to our hospital due to “chest and back pain for 7 hours.” Enhanced computed tomography (CT) of the aorta revealed a Standford A dissection, involving 3 branches of the aortic arch and the opening of the right coronary artery. The aortic root and ascending aorta were significantly widened.

**Diagnoses:**

Acute type A aortic dissection.

**Interventions:**

After multidisciplinary discussions, we decided to perform cesarean section first and then cardiac surgery. A live male infant was delivered successfully by Obstetrician and Gynecologist. And then, we performed the Betalls procedure with the use of a mechanical 23# aortic-valve vessel for the patient. The innominate artery openings were reinforced with felt pads.

**Outcomes:**

The procedure was successful. CT examination at 2 months after operation showed that the true lumen of the aorta was enlarged, and no dissection was found in the 3 branches of the aortic arch.

**Lessons:**

Type A aortic dissection during pregnancy is a rare event that carries a high risk of death for both mother and fetus. An optimal outcome can be achieved through early and accurate diagnosis, safe imaging techniques, timely and effective multidisciplinary discussion, and precise and individualized treatment.

## 1. Introduction

Acute type A aortic dissection is a rare and catastrophic complication of pregnancy with a very high mortality rate for both the mother and the fetus. It usually occurs during the third trimester or puerperium. Once such lesions occur, early detection, multidisciplinary discussion and timely and effective treatment are critical to maternal and fetal prognosis. Herein, we successfully report a case of acute type A aortic dissection at 31 weeks of gestation.

## 2. Case report

**Figure 1.**
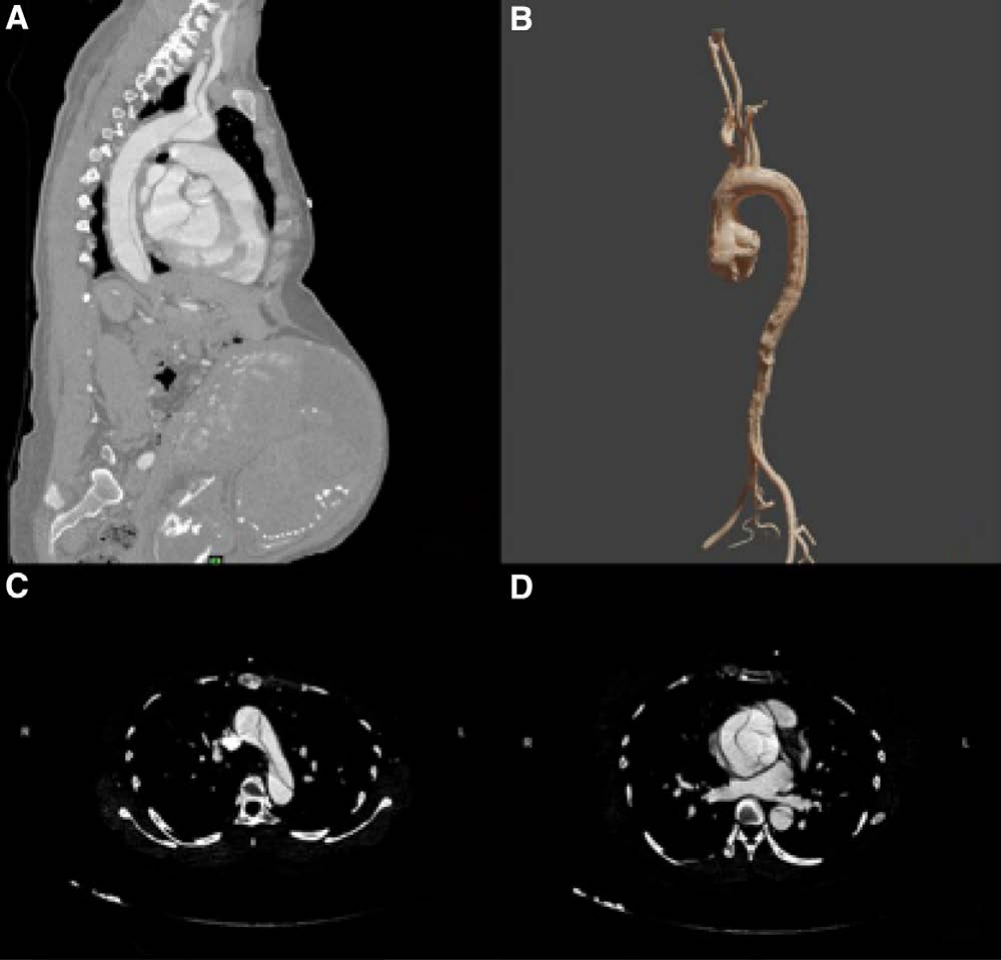
Preoperative CT showed a Standford A dissection, involving 3 branches of the aortic arch (A, B); The ascending aorta and aortic root were significantly widened the aortic valve is involved (C, D). CT = computed tomography.

A 40-year-old female who was 31 weeks pregnant was transferred from a local hospital to the emergency department of our hospital due to “chest and back pain for 7 hours.” She had a history of cesarean delivery 6 years ago, and had no history of fever, hypertension, connective tissue disease, or congenital heart disease. Physical examination after admission showed: Temperature: 37°C, Heart rate: 83 beats/min, Respiration: 12 beats/min, Blood pressure: 120/41 mm Hg. Laboratory results showed that white blood cell count 19 × 10^9^/L, N% 92.3, C-reactive protein 7.6 mg/L. Electrocardiograph indicated a sinus rhythm. Emergency bedside echocardiography showed that the sinus of the aorta widened about 55 mm, and streaming-like echo could be seen in the ascending aorta. Enhanced computed tomography (CT) of the aorta revealed a Standford A dissection, involving 3 branches of the aortic arch and the opening of the right coronary artery. The aortic root and ascending aorta were significantly widened. The celiac trunk, superior and inferior mesenteric arteries and bilateral renal arteries all originated from the true lumen. Single fetus is seen in utero (Fig. 1).

After admission, we carried out dynamic monitoring of the mother and fetus, and urgently organized multidisciplinary discussions including cardiac surgery, obstetrics and gynecology, anesthesiology, and critical care medicine. Considering that extracorporeal circulation may have adverse effects on the fetus, we finally decided to perform cesarean section first and then cardiac surgery. A live male infant was delivered successfully under general anesthesia and immediately transferred to the neonatal care unit. Apgar scores of 1 and 5 minutes were 6 and 8, respectively. 20U oxytocin was injected into the fundus muscle, the abdominal incision was sutured and the uterine cavity was filled with gauze, and the bleeding was closely monitored.

**Figure 2.**
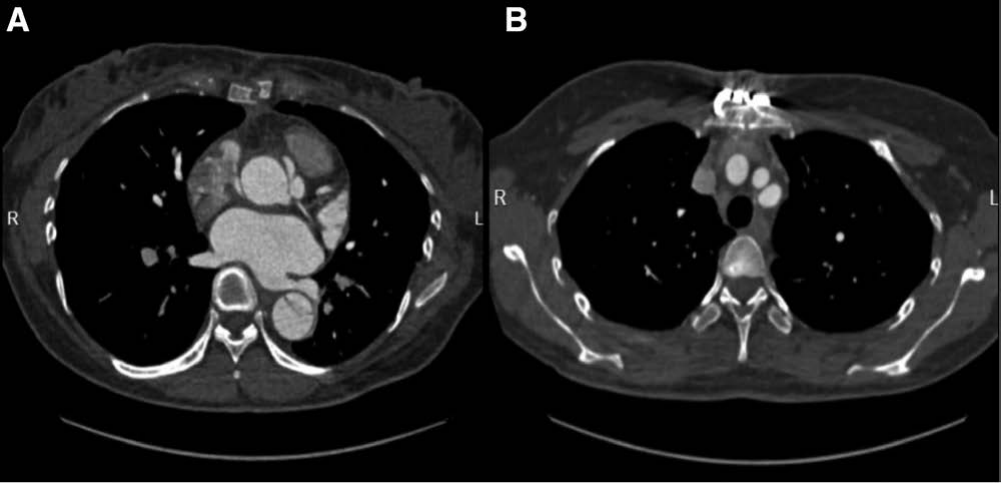
CT examination at 2 months after operation showed that the true lumen of the aorta was enlarged, and no dissection was found in the 3 branches of the aortic arch. CT = computed tomography.

After the cesarean section, the median sternal incision was selected, and cardiopulmonary bypass was established through the right femoral artery intubation and a dual stage venous cannula after heparinization. Left ventricle venting tube and retrograde cardioplegia catheter were placed. Circulatory arrest was instituted when the nasopharyngeal temperature reached 25°C. After cardiac arrest by retrograde perfusion of cardioplegia (Del Nido), it was found that the sinus of the ascending aorta was widened by about 55 mm, and the diameter of the ascending aorta was significantly widened through aortic incision. The dissection tear was located in the ascending aorta, with a diameter of about 2 cm. Poor aortic valve alignment resulted in severe regurgitation. On the basis of the involvement of the aortic valve, aortic sinus, and ascending aorta, but the mild involvement of the 3 branches of the aortic arch, we performed the Betalls procedure with the use of a mechanical 23# aortic-valve vessel. The innominate artery openings were reinforced with felt pads. The total cardiopulmonary bypass time was 210 minutes. The aortic cross-clamp time were 175 minutes. The operation was successful, and the tracheal tube was extubated on the second day after surgery. The intrauterine gauze was removed when there was no bleeding on the third day after operation. The patient recovered well after operation and was discharged successfully. CT examination at 2 months after operation showed that the true lumen of the aorta was enlarged, and no dissection was found in the 3 branches of the aortic arch (Fig. 2).

## 3. Discussion

Aortic dissection is a rare and fatal complication in pregnant women. The incidence varies from 0.4 to 3.5 cases per 100,000 person-years, and the overall mortality can be as high as 60%.^[1]^ Aortic dissection is a rare and fatal complication in pregnant women. It is more commonly secondary to inherited connective-tissue disease, valve abnormalities, and vascular inflammation, all of which predispose to aortic disease but have also been reported in patients without a genetic history.^[2,3]^ Approximately 50% of dissection during pregnancy occurs in women younger than 40 years of age, usually in the third trimester or early postpartum period.^[4]^ The mechanisms of aortic dissection during pregnancy are still unclear. Some studies have shown that it may be due to the increase of maternal heart rate, stroke volume, cardiac output, left ventricular wall mass and end diastolic size, which leads to the tear of internal arterial veins.^[5,6]^ In addition, estrogen and progesterone cause histological structural changes in the aortic wall during the first trimester, reshaping the media and intima. The breakdown of reticular fibers, the reduction of acidic mucopolysaccharides, and the loss of elastic fiber ripple in the blood vessels contribute to an increased risk of dissection.^[7,8]^ Therefore, a better understanding of the underlying mechanisms that cause aortic dissection during pregnancy and in the early postpartum period may help determine timely treatment options. In addition to routine fetal monitoring and prenatal care during pregnancy, it is also important to pay close attention to maternal echocardiographic indicators.

The symptoms of aortic dissection are usually sudden chest and back pain, syncope, vomiting, sweating and so on. Therefore, attention should be paid to these symptoms in pregnant women. The diagnosis methods mainly include echocardiography, chest X-ray, enhanced aortic CT and aortography. Echocardiography is the best choice for the diagnosis of aortic valve disease. However, enhanced aortic CT is an excellent diagnostic tool for establishing the type of aortic dissection and for determining further management, despite ethical concerns about radiation dose and the effect of intravenous contrast material on pregnant patients. Studies have shown that the potential risk of cancer caused by CT examination is relatively low, especially in the third trimester, between 1.5% and 2.0%.^[9]^ In our case, considering the critical condition of the patient in the third trimester of pregnancy, we decided to perform an enhanced CT scan after communication with the patient, which provided sufficient evidence for the diagnosis and further management.

The treatment of pregnancy complicated with acute type A aortic dissection still faces many challenges, and patients with different conditions need individualized treatment. Nevertheless, the challenges of performing surgery and a management strategy with cardiopulmonary bypass place the fetus at high risk, and therefore the choice of treatment must be careful. One of the problems we are facing is when to deliver the baby. The European Society of Cardiology guidelines recommend delivery at 26 weeks of gestation when fetal maturity is likely to have been reached.^[10]^ Zeebregts et al^[11]^ also suggested that patients with a gestation of more than 32 weeks should undergo cesarean section followed by aortic repair. For patients with a gestation of <28 weeks, the dissection should be surgically repaired and the pregnancy continued until the fetus is mature. Between 28 and 32 weeks of gestation, the approach is dictated by the condition of the fetus, although Zhu et al^[12]^ demonstrate adequate survival rates with delivery-first approach after 28 gestational weeks. In our case, the patient was a senile gravida who had reached 31 weeks of gestation and was critically ill on admission. Fetal monitoring after admission indicated that the fetus was mature, with stable vital signs and no symptoms of fetal distress. Through multidisciplinary consultation, it is recommended to perform fetal delivery under the premise of maintaining stable vital signs of the patient, so as to avoid the influence of hypotension and heparinization on the uterus and placenta during cardiopulmonary bypass, thus increasing the survival rate of the fetus. Another problem is the choice of aortic surgery. There is a substantial debate over whether to limit the extent of aortic surgery to save the patient with limited aortic approach or to expand the procedure more aggressively. If only the ascending aorta is involved, the surgeon can only consider hemiarch replacement. However, if the aortic arch is involved, the choice of surgical method should consider whether the elephant trunk stent technique is needed.^[13]^ In this case, the aortic dissection tear was located in the ascending aorta, the aortic sinus and aortic valve were involved, the innominate artery opening was slightly involved, the left common carotid artery and the left subclavian artery were almost not involved, so we only performed the conventional Betall procedure on the patient. Innominate artery openings were reinforced with Prolene sutures with felt sheets. This approach minimizes the operation time while resolving the patient lesions. At the same time, we adopted the high-flow, high-pressure and deep hypothermia cardiopulmonary bypass to ensure the perfusion of important organs and reduce the oxygen consumption of organs. The patient uneventful recovery confirmed the feasibility of our procedure. Of course, for patients with severe aortic arch and descending aorta disease, total aortic arch replacement combined with elephant trunk stenting technique can maximize the relief of the disease and improve the prognosis.

## 4. Conclusion

Type A aortic dissection during pregnancy is a rare event that carries a high risk of death for both mother and fetus. An optimal outcome can be achieved through early and accurate diagnosis, safe imaging techniques, timely and effective multidisciplinary discussion, and precise and individualized treatment.

## Author contributions

**Conceptualization:** Fan He.

**Methodology:** Shiqiang Wang.

**Supervision:** Bijun Xu.

**Writing – original draft:** Jianmao Hong.

**Writing – review & editing:** Yue Wang.
